# Supplementary figures and images for: EBV Associated Breast Cancer Whole Methylome Analysis Reveals Viral and Developmental Enriched Pathways
Source: Front Oncol. 2018 Aug 13;8:316. doi: 10.3389/fonc.2018.00316 (PMC6099083; doi:10.3389/fonc.2018.00316)

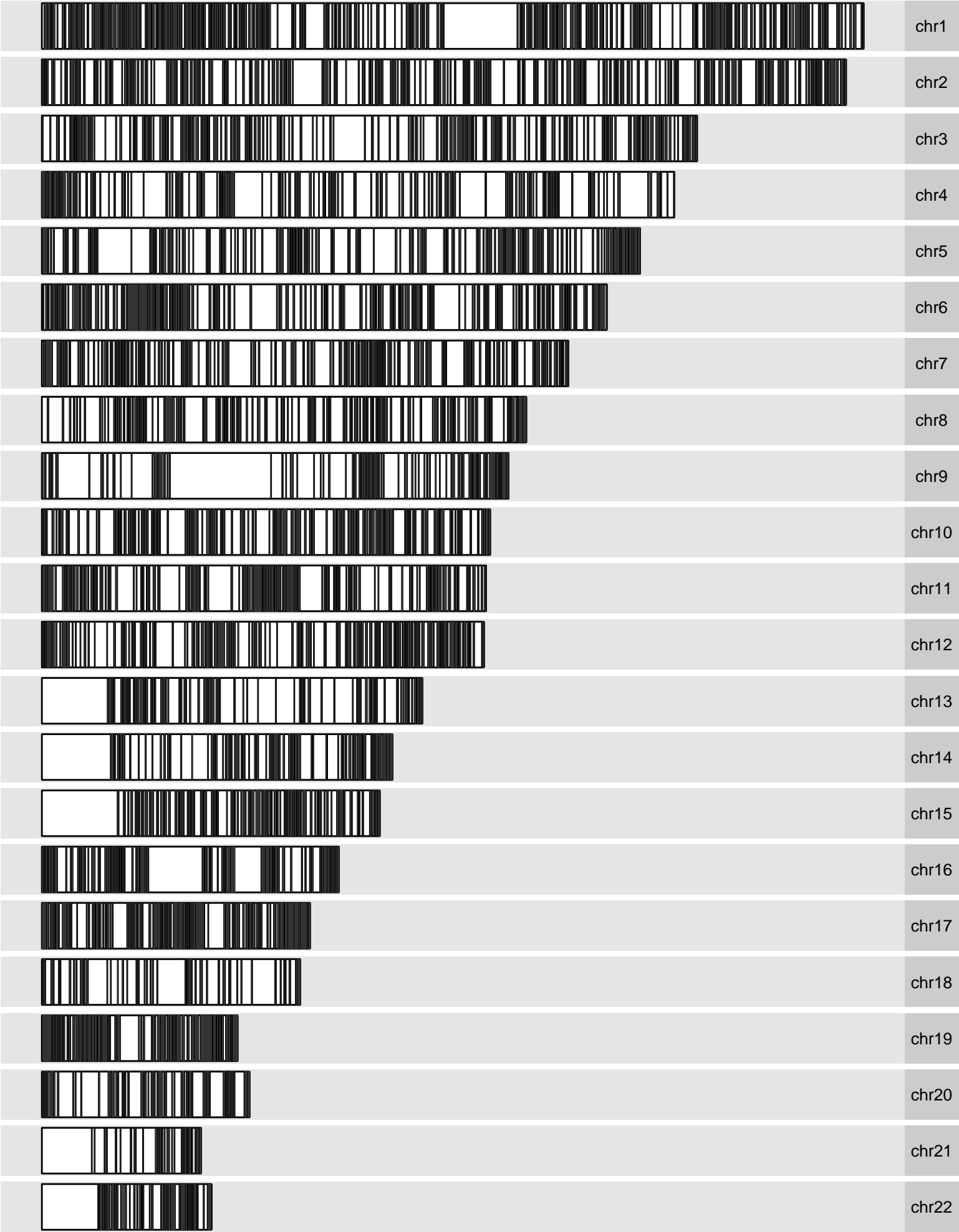

0 Mb 50 Mb 100 Mb 150 Mb 200 Mb 250 Mb

Supplement: Figure S1 — Genomic distribution of hypermethylation marks shown at each chromosome. Black color indicates hypermethylation sites. [file Image_1.pdf]

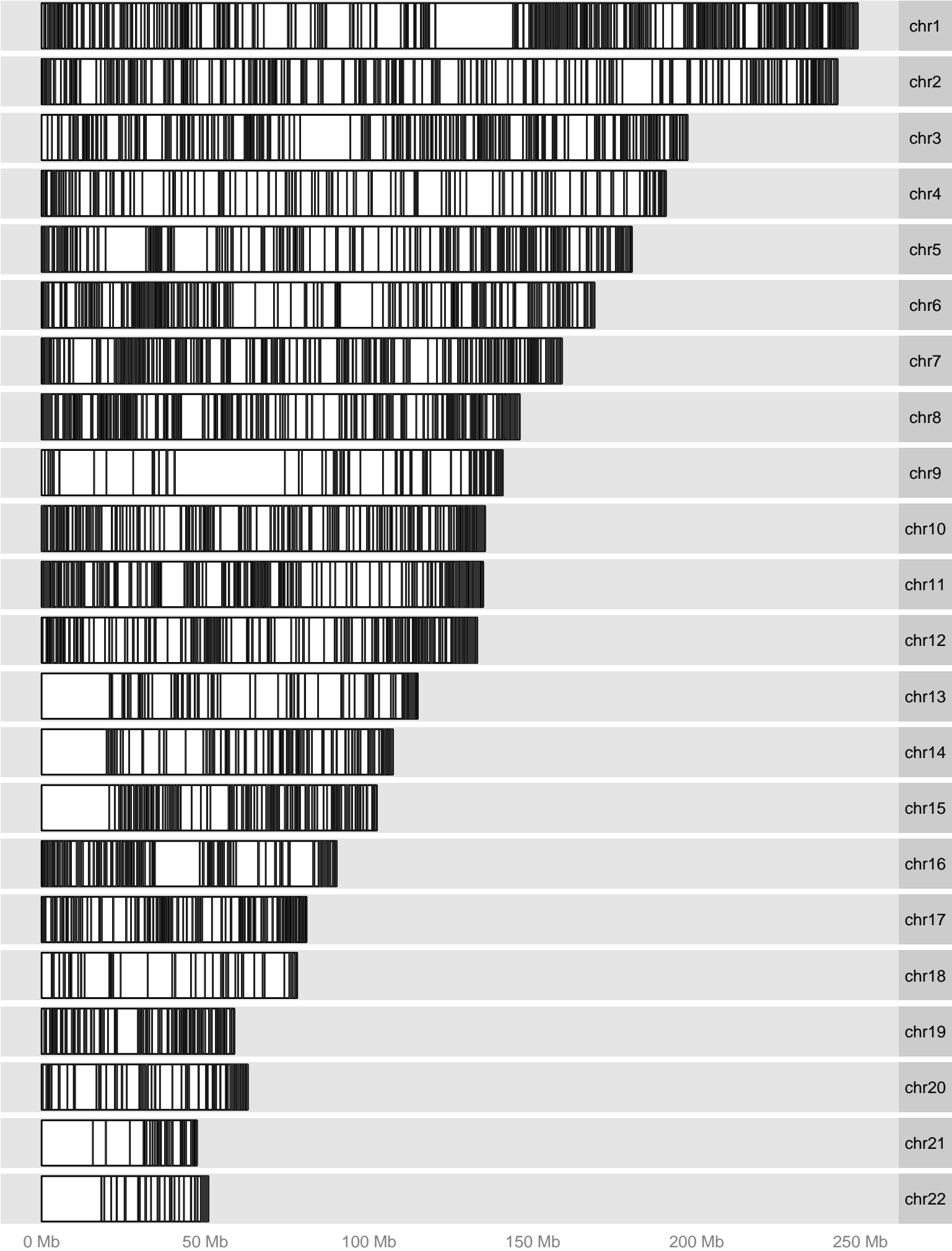

Supplement: Figure S2 — Genomic distribution of hypomethylation marks shown at each chromosome. Black color indicates hypermethylation sites. [file Image_2.pdf]

Beta-value distribution (463440 array sites)

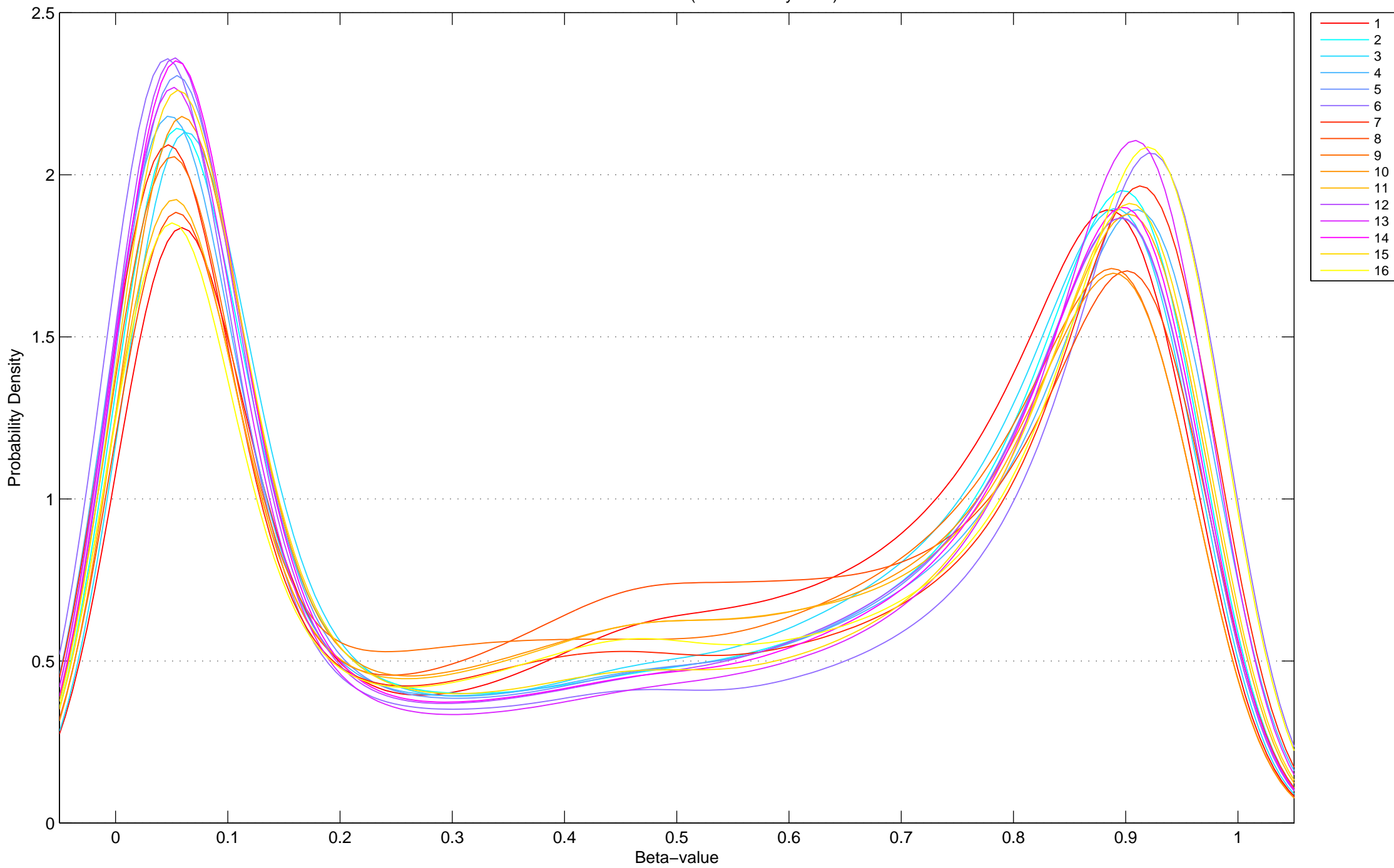

Supplement: Figure S3 — Density plots of beta values for individual samples. Shades of red and yellow colors represent tumor samples, whereas shades of blue and green represent normal samples. [file Image_3.pdf]
