# Supplementary material for: EBV Associated Breast Cancer Whole Methylome Analysis Reveals Viral and Developmental Enriched Pathways
Source: Front Oncol. 2018 Aug 13;8:316. doi: 10.3389/fonc.2018.00316 (PMC6099083; doi:10.3389/fonc.2018.00316)
Supplement: Table S1 — Pathway enrichment results for breast cancer related Epigenetically Downregulated Genes (EDG) subnetwork. ReactomeFI cytoscape app was used to extract breast cancer related subnetworks from EUG set by loading NCI cancer index and performing pathway enrichment analysis on interaction networks. Nodes that corresponded to malignant breast cancer were selected. The table shows the enriched pathways, the number of genes in the pathway from the total query gene set, and the number of genes in the pathway found in the interaction network. Results having p-values < 0.01 and a False Detection Rate < 0.001 are shown. [file Table_1.docx]

| **Pathway** | **Number of genes in the Geneset** | **Number of genes in the Network** | **FDR** |
| --- | --- | --- | --- |
| PI3K-Akt signaling pathway(K) | **346** | **45** | **<1.000e-03** |
| Pathways in cancer(K) | **327** | **43** | **<5.000e-04** |
| Proteoglycans in cancer(K) | **225** | **32** | **<3.333e-04** |
| Ras signaling pathway(K) | **227** | **30** | **<2.500e-04** |
| Prolactin signaling pathway(K) | **72** | **17** | **<2.000e-04** |
| Rap1 signaling pathway(K) | **213** | **28** | **<1.667e-04** |
| Angiopoietin receptor Tie2-mediated signaling(N) | **50** | **14** | **<1.429e-04** |
| Focal adhesion(K) | **206** | **26** | **<1.250e-04** |
| HTLV-I infection(K) | **260** | **29** | **<1.111e-04** |
| Signaling by Type 1 Insulin-like Growth Factor 1 Receptor (IGF1R)(R) | **86** | **16** | **<1.000e-04** |
| Hepatitis B(K) | **146** | **20** | **<9.091e-05** |
| Signaling by ERBB4(R) | **147** | **20** | **<8.333e-05** |
| Signalling by NGF(R) | **279** | **28** | **<7.692e-05** |
| SHP2 signaling(N) | **51** | **12** | **<7.143e-05** |
| GPCR ligand binding(R) | **433** | **36** | **<6.667e-05** |
| Signaling events mediated by VEGFR1 and VEGFR2(N) | **63** | **13** | **<6.250e-05** |
| IL2-mediated signaling events(N) | **54** | **12** | **<5.882e-05** |
| Rheumatoid arthritis(K) | **90** | **15** | **<5.556e-05** |
| HIF-1-alpha transcription factor network(N) | **66** | **13** | **<5.263e-05** |
| MAPK signaling pathway(K) | **259** | **26** | **<5.000e-05** |
| Osteoclast differentiation(K) | **132** | **18** | **<4.762e-05** |
| FGF signaling pathway(P) | **92** | **15** | **<4.545e-05** |
| Measles(K) | **134** | **18** | **<4.348e-05** |
| Regulation of actin cytoskeleton(K) | **215** | **23** | **<4.167e-05** |
| Jak-STAT signaling pathway(K) | **156** | **19** | **<4.000e-05** |
| Chronic myeloid leukemia(K) | **73** | **13** | **<3.846e-05** |
| Viral carcinogenesis(K) | **206** | **22** | **<3.704e-05** |
| MicroRNAs in cancer(K) | **296** | **27** | **<3.571e-05** |
| Signaling by PDGF(R) | **176** | **20** | **<3.448e-05** |
| Signaling events mediated by PTP1B(N) | **52** | **11** | **<3.333e-05** |
| Pancreatic cancer(K) | **66** | **12** | **<3.226e-05** |
| Signaling events mediated by Hepatocyte Growth Factor Receptor (c-Met)(N) | **80** | **13** | **<3.125e-05** |
| EPHA2 forward signaling(N) | **18** | **7** | **<3.030e-05** |
| Signaling by SCF-KIT(R) | **137** | **17** | **<2.941e-05** |
| Non-small cell lung cancer(K) | **56** | **11** | **<2.857e-05** |
| Signaling by Insulin receptor(R) | **109** | **15** | **<2.778e-05** |
| FGF signaling pathway(N) | **46** | **10** | **<2.703e-05** |
| Signaling by EGFR(R) | **171** | **19** | **<2.632e-05** |
| Adipocytokine signaling pathway(K) | **70** | **12** | **<2.564e-05** |
| Integrins in angiogenesis(N) | **47** | **10** | **<2.500e-05** |
| GMCSF-mediated signaling events(N) | **37** | **9** | **<2.439e-05** |
| Melanoma(K) | **71** | **12** | **<2.381e-05** |
| Cell adhesion molecules (CAMs)(K) | **143** | **17** | **<2.326e-05** |
| IL2 signaling events mediated by STAT5(N) | **28** | **8** | **<2.273e-05** |
| p73 transcription factor network(N) | **73** | **12** | **<2.222e-05** |
| VEGF signaling pathway(K) | **61** | **11** | **<2.174e-05** |
| Fc-epsilon receptor I signaling in mast cells(N) | **62** | **11** | **<2.128e-05** |
| ErbB signaling pathway(K) | **88** | **13** | **<2.083e-05** |
| IGF1 pathway(N) | **30** | **8** | **<2.041e-05** |
| Leukocyte transendothelial migration(K) | **118** | **15** | **<2.000e-05** |
| Ras Pathway(P) | **63** | **11** | **<1.923e-05** |
| IL4-mediated signaling events(N) | **63** | **11** | **<1.923e-05** |
| Neurotrophin signaling pathway(K) | **120** | **15** | **<1.852e-05** |
| PDGFR-beta signaling pathway(N) | **120** | **15** | **<1.852e-05** |
| Interleukin-2 signaling(R) | **42** | **9** | **3.64E-05** |
| Signaling by FGFR(R) | **154** | **17** | **3.51E-05** |
| Hippo signaling pathway(K) | **154** | **17** | **3.51E-05** |
| Syndecan-2-mediated signaling events(N) | **32** | **8** | **3.45E-05** |
| Angiogenesis(P) | **66** | **11** | **3.33E-05** |
| Renal cell carcinoma(K) | **66** | **11** | **3.33E-05** |
| Signaling by ERBB2(R) | **155** | **17** | **3.28E-05** |
| Chemokine signaling pathway(K) | **189** | **19** | **3.23E-05** |
| TNF signaling pathway(K) | **110** | **14** | **6.35E-05** |
| Cytokine-cytokine receptor interaction(K) | **265** | **23** | **6.25E-05** |
| Alpha9 beta1 integrin signaling events(N) | **24** | **7** | **6.15E-05** |
| Fc epsilon RI signaling pathway(K) | **70** | **11** | **7.46E-05** |
| AP-1 transcription factor network(N) | **70** | **11** | **7.46E-05** |
| PIP3 activates AKT signaling(R) | **98** | **13** | **7.35E-05** |
| Glypican 1 network(N) | **25** | **7** | **7.25E-05** |
| Signaling events mediated by TCPTP(N) | **35** | **8** | **7.04E-05** |
| IL2 signaling events mediated by PI3K(N) | **35** | **8** | **7.04E-05** |
| Direct p53 effectors(N) | **133** | **15** | **1.25E-04** |
| Epstein-Barr virus infection(K) | **202** | **19** | **1.23E-04** |
| Hematopoietic cell lineage(K) | **88** | **12** | **1.35E-04** |
| Malaria(K) | **49** | **9** | **1.60E-04** |
| Prostate cancer(K) | **89** | **12** | **1.58E-04** |
| T cell receptor signaling pathway(K) | **104** | **13** | **1.69E-04** |
| Colorectal cancer(K) | **62** | **10** | **1.67E-04** |
| Extracellular matrix organization(R) | **263** | **22** | **2.28E-04** |
| DAP12 interactions(R) | **171** | **17** | **2.38E-04** |
| BARD1 signaling events(N) | **29** | **7** | **3.05E-04** |
| Thyroid cancer(K) | **29** | **7** | **3.05E-04** |
| Endometrial cancer(K) | **52** | **9** | **3.13E-04** |
| Glioma(K) | **65** | **10** | **3.10E-04** |
| Cell surface interactions at the vascular wall(R) | **95** | **12** | **3.65E-04** |
| T cell activation(P) | **81** | **11** | **4.19E-04** |
| Signaling by Leptin(R) | **21** | **6** | **4.37E-04** |
| Plasma membrane estrogen receptor signaling(N) | **42** | **8** | **4.43E-04** |
| EGF receptor signaling pathway(P) | **82** | **11** | **4.72E-04** |
| p53 signaling pathway(K) | **68** | **10** | **4.78E-04** |
| Beta3 integrin cell surface interactions(N) | **43** | **8** | **4.95E-04** |
| p53 pathway(P) | **44** | **8** | **5.76E-04** |
| Acute myeloid leukemia(K) | **57** | **9** | **6.02E-04** |
| Progesterone-mediated oocyte maturation(K) | **86** | **11** | **6.49E-04** |
| ATF-2 transcription factor network(N) | **58** | **9** | **6.63E-04** |
| Inflammation mediated by chemokine and cytokine signaling pathway(P) | **72** | **10** | **6.67E-04** |
| Natural killer cell mediated cytotoxicity(K) | **135** | **14** | **6.60E-04** |
| Gastrin-CREB signalling pathway via PKC and MAPK(R) | **207** | **18** | **6.53E-04** |
| Toxoplasmosis(K) | **119** | **13** | **6.97E-04** |
| Costimulation by the CD28 family(R) | **74** | **10** | **7.70E-04** |
| Coregulation of Androgen receptor activity(N) | **60** | **9** | **7.62E-04** |
| Interleukin-3, 5 and GM-CSF signaling(R) | **47** | **8** | **7.55E-04** |
| Wnt signaling pathway(K) | **139** | **14** | **8.06E-04** |
| HIF-1 signaling pathway(K) | **106** | **12** | **8.27E-04** |
| VEGFR3 signaling in lymphatic endothelium(N) | **25** | **6** | **8.29E-04** |
| Trk receptor signaling mediated by PI3K and PLC-gamma(N) | **36** | **7** | **8.77E-04** |
| NF-kappa B signaling pathway(K) | **91** | **11** | **8.88E-04** |
| Signaling events mediated by focal adhesion kinase(N) | **62** | **9** | **9.26E-04** |
| Glucocorticoid receptor regulatory network(N) | **77** | **10** | **9.82E-04** |

**Table S1 Pathway enrichment results for EDG breast subnetwork**
